# Association between thoracolumbar kyphosis and fracture risk assessment tool in Korean postmenopausal women: A retrospective study

**DOI:** 10.1371/journal.pone.0358889

**Published:** 2026-10-05

**Authors:** Tae Sik Goh, Yun Kyung Jeon, Kyoungjune Pak, Jihyun Kim, Keunyoung Kim

**Affiliations:** 1 Department of Orthopaedic Surgery, Biomedical Research Institute, Pusan National University Hospital, Pusan National University School of Medicine, Busan, Republic of Korea; 2 Department of Internal Medicine and Biomedical Research Institute, Pusan National University Hospital, Busan, Republic of Korea; 3 Department of Internal Medicine, School of Medicine, Pusan National University, Yangsan, Republic of Korea; 4 Department of Nuclear Medicine and Biomedical Research Institute, Pusan National University Hospital, Busan, Republic of Korea; 5 Department of Nuclear Medicine, School of Medicine, Pusan National University, Yangsan, Republic of Korea; University of Life Sciences in Lublin, POLAND

## Abstract

Thoracolumbar kyphosis (TLK) has been suggested as a potential risk factor for vertebral fractures in postmenopausal women; however, its association with bone mineral density (BMD), trabecular bone score (TBS), and fracture risk assessment tools, such as the Fracture Risk Assessment Tool (FRAX), remain undefined. In this study, we investigated the association between the TLK level and fracture risk in postmenopausal women. We retrospectively analyzed the associations between the thoracolumbar Cobb (TLCobb) angle, baseline compression fractures, BMD, TBS, and volumetric BMD measured using dual-energy X-ray absorptiometry (DXA) and central quantitative computed tomography (cQCT). The study included 251 postmenopausal women (median age, 61 years; interquartile range, 56–69 years) who visited our hospital for health checkups between March 2020 and December 2021. Fracture risk was assessed using FRAX and TBS-adjusted FRAX. Baseline radiography revealed patients with compression fractures (n = 64, 25.498%), in whom the TLCobb angle was significantly higher. The TLCobb angle demonstrated a significant negative correlation with TBS. Baseline compression fractures and higher TLCobb angles were significant predictors of future fragility compression fractures, with a TLCobb angle > 9.4° established as the threshold value. DXA and FRAX had limitations in predicting future progressive or new-onset compression fractures. In conclusion, TLK was found to be associated with densitometric results, particularly TBS, and has predictive value for future compression fracture risk. Baseline compression fractures with higher TLCobb angles are potential predictors of fracture risk in postmenopausal women.

## Introduction

The thoracolumbar junction, from T11 to L2, represents a critical transition zone, being the most common site of vertebral compression fractures, particularly in individuals with osteoporosis [1,2]. Biomechanically, this region serves as a structural weak point, bridging the rigid kyphotic thoracic spine with the more mobile lordotic lumbar spine [3]. The thoracic spine, which is characterized by physiological kyphosis, maintains significant intrinsic stability because of the structural support provided by the ribs and sternum. In contrast, the lumbar spine, which is characterized by physiological lordosis, exhibits a wide range of flexion and extension, a function enabled by the sagittal orientation of the joint facets. Although thoracolumbar kyphosis (TLK) is widely acknowledged as a contributing factor to vertebral compression fractures, the exact degree of kyphosis, quantified using the Cobb angle, which confers an elevated clinical risk and necessitates therapeutic intervention, remains unclear.

Osteoporosis frequently occurs in older adults and is commonly associated with falls and fractures, leading to higher morbidity and mortality rates [4–6], as well as increased dependence in older adults [7,8]. Therefore, accurate prediction of osteoporotic fractures is crucial in clinical settings. In osteoporosis, bone strength decreases owing to the deterioration of bone quality and loss of bone mass associated with aging.

Currently, the diagnosis of osteoporosis is mainly based on bone mineral density (BMD) measured using dual-energy X-ray absorptiometry (DXA) [9]. Lower T-scores are associated with an increased likelihood of osteoporotic fractures. Although the T-score is a key measure for diagnosing osteoporosis, it cannot reliably predict osteoporotic fractures [10,11]. Indeed, most osteoporotic fragility fractures occur in osteopenia rather than osteoporosis [12], which might be because the diagnosis of osteoporosis is based on bone mass and does not account for bone quality.

Recently, imaging modalities such as the trabecular bone score (TBS) and central quantitative computed tomography (cQCT) have been increasingly employed in clinical settings to address the limitations of DXA and provide a more accurate assessment of bone health. The TBS, derived from texture analysis of DXA images, reflects the strength of the trabecular microarchitecture [13]. In contrast, cQCT offers the advantage of excluding factors such as spinal stenosis and aortic calcification, which can lead to an overestimation of BMD using DXA by directly measuring the volumetric BMD (vBMD) at central sites such as the lumbar spine (LS) and hip [14]. However, few studies have investigated the association between bone quality and TLK levels when assessing bone health.

Therefore, we aimed to investigate fracture risk prediction using the Fracture Risk Assessment Tool (FRAX) in relation to TLK, baseline compression fractures, TBS, and other BMD values in postmenopausal women.

## Materials and methods

### Study population

This retrospective study included postmenopausal women who voluntarily visited our outpatient clinic for bone-health screening between March 1, 2020 and December 31, 2021. All clinical and imaging data were obtained from examinations performed as part of routine clinical care, and no additional examinations were performed for research purposes. DXA and baseline thoracolumbar spine radiography are routinely performed in this setting, whereas cQCT is performed as an additional examination when clinically indicated or when patients agree to further volumetric BMD assessment. Women with complete DXA, cQCT, and baseline radiographic data were retrospectively identified for the present analysis. The exclusion criteria were as follows: abnormal thyroid-stimulating hormone levels; history of malignancy; and other underlying diseases affecting bone status, including diabetes mellitus, rheumatic arthritis, and hepatitis. This retrospective study was approved by our Institutional Review Board, which waived the requirement for written informed consent (IRB No. 2002-011-088).

### DXA

The areal BMD (aBMD) values of the LS, femoral neck (FN), and total hip (TH) were determined using DXA (Lunar Prodigy; GE Medical Systems, Madison, WI, USA). Daily calibration and quality assurance testing were performed, and the coefficient of variation for precision in measuring aBMD was 0.34%. The aBMD was expressed as g/cm^2^, and the T-score was defined as the standard deviation (SD) from the mean BMD of a reference group from the general population, matched for sex and aged 25–35 years, analyzed using Encore software (version 13.0; GE Medical Systems). The diagnosis was based on the WHO classification using the T-score, as follows: T-score ≤ –2.5 SD was defined as osteoporosis, T-score between –2.5 and –1.0 SD was defined as osteopenia, and T-score ≥ –1.0 SD was considered normal. The TBS at the LS was generated from the LS DXA file using the TBS iNsight software (version 3.0.2.0; Medimaps, Geneva, Switzerland). We used the following TBS cutoffs proposed by an international working group of TBS users for postmenopausal women: normal status at values ≥ 1.35, partially degraded microarchitectural status at values between 1.20–1.35, and degraded microarchitectural status at values < 1.20 [13].

### cQCT

To calculate the vBMD (mg/cm^3^) at the LS, cQCT was performed using a Philips Brilliance 16-slice multidetector helical CT scanner (GEMINI TF CT, Philips, Eindhoven, The Netherlands) at a voltage of 120 kVp, with a slice thickness of 3 mm, in conjunction with a solid-state CT calibration phantom (Model 3 QA phantom, Mindways software). Daily quality assurance was performed, and the coefficient of variation for precision in measuring the vBMD at the LS was 0.34%. The vBMD was measured only at L1–L3 due to the limited scanning field to minimize radiation exposure and improve spatial resolution. The simultaneously scanned CT images of the patients and calibration phantom were processed using QCT Pro (version 4.2.3., Mindways Software, Inc., Austin, TX, USA). The diagnostic categorization recommended by the American College of Radiology was applied as follows: vBMD < 80 mg/cm^3^, osteoporosis; vBMD between 80 and 120 mg/cm^3^, osteopenia; and vBMD > 120 mg/cm^3^, normal.

### X-ray protocol and calculation of Cobb angle

Radiographic assessment of the lateral view of the thoracolumbar spine was performed to diagnose fractures, assess spinal alignment, evaluate degenerative changes, and detect underlying pathology. Images were acquired in a standing neutral position, avoiding excessive flexion or extension, with T11–L2 centered on the X-ray detector. The severity of TLK was assessed by measuring the sagittal thoracolumbar Cobb (TLCobb) angle on baseline radiographs. The TLCobb angle was measured by drawing lines along the superior endplate of T10 and the inferior endplate of L2, and then extending perpendicularly from each; the angle at their intersection represented the TLCobb angle. In addition, predisposing compression fractures, defined according to the Genant criteria, were identified by reviewing radiographs [15].

### Calculation of FRAX probabilities

The 10-year probability of major osteoporotic and hip fractures was calculated for each woman using an online algorithm available at http://shef.ac.uk/FRAX, based on clinical variables: age, body mass index, previous fractures, parental history of hip fracture, alcohol use, smoking status, systemic glucocorticoid use, rheumatoid arthritis, secondary osteoporosis, and total hip BMD. The TBS-adjusted 10-year probabilities were calculated using an online algorithm available at http://www.shef/ac.uk/TBS/Calculation_Tool.aspx.FRAX and TBS-adjusted FRAX scores were calibrated according to the South Korean National Epidemiological Database [16].

### Follow-up protocol

All patients regularly visited the hospital for follow-up according to the protocol developed based on the results of physical examinations, history taking, and radiography of the spine. At our institution, postmenopausal women undergoing osteoporosis screening are generally followed up on an annual basis. Patients with normal findings are typically followed through the health check-up center, whereas those with abnormal findings are referred to the endocrinology clinic for further evaluation and management. Follow-up data were retrospectively obtained from electronic medical records. Patients with incomplete or missing key clinical or imaging data were excluded during the initial patient selection process to ensure data completeness and reliability.

The primary endpoint of the analysis was fragility fracture-free survival, defined as the time interval between the date of baseline imaging and the detection of a progressive or new-onset of structural compression fracture. All fragility compression fractures were defined according to the Genant criteria [15].

### Statistical analysis

Values of all normally distributed variables are expressed as the mean ± SD, whereas values of non-normally distributed variables are expressed as medians and interquartile ranges (IQR; 25%–75%). *Chi-square* test was used to compare categorical data of different groups. Pearson’s correlation coefficient (*r*) was calculated to analyze associations between variables using linear regression analyses. The TLCobb threshold for predicting progressive compression fractures was determined using a receiver operating characteristic (ROC) curve. Fracture-free survival curves were plotted using the Kaplan–Meier method, and the log-rank test was used to determine significance. Cox proportional hazards regression analysis was performed to assess the association between various covariates and fracture-free survival using the stepwise selection method to identify the most significant predictors of hazard. Statistical significance was set at *p* = 0.01 for both entry and removal of variables to ensure more stringent criteria. Statistical analyses were performed using MedCalc software (version 16.4.3; MedCalc, Mariakerke, Belgium) and RStudio (version 1.2b; Rstudio, MA, USA). Statistical significance was set at *p* < 0.05.

## Results

### Baseline characteristics

Two hundred and fifty-one patients, with a median age of 61 years (IQR: 56–69 years) were included in the study. Baseline fragility compression fractures were identified in 64 patients (25.5%), and the median TLCobb angle was 7.2° (IQR: 3.700–12.800). Based on the lowest T-scores across the LS, FN, and TH sites, 75 patients (29.9%) were classified as normal, 132 (52.6%) as osteopenic, and 44 (17.5%) as osteoporotic. The distribution of BMD categories at each anatomical site was as follows: at the LS, 118 patients (47.0%) were classified as normal, 106 (42.2%) as osteopenic, and 27 (10.8%) as osteoporotic; at the FN, 111 patients (44.2%) were classified as normal, 114 (45.4%) as osteopenic, and 26 (10.4%) as osteoporotic; at the TH, 144 patients (57.4%) were classified as normal, 86 (34.3%) as osteopenic, and 21 (8.4%) as osteoporotic. Among all participants, 81 (32.3%) had a normal TBS. Base on cQCT, approximately half of the participants (n = 121, 48.2%) had osteoporotic vBMD (Table 1). TBS and cQCT classified a greater proportion of participants as having impaired bone status than did DXA-based BMD assessment (*p* < 0.001).

**Table 1:** Baseline clinical and densitometric characteristics of the Korean postmenopausal study population.

	N = 251
Age (years)	61.0 [56.0, 69.0]
Gender (Female)	251 (100%)
Ethnicity (Korean)	251 (100%)
Follow-up period (month)	20.000 [12.000;32.000]
Height	157.000 [153.000;160.000]
Weight	57.000 [52.000;63.000]
BMI	20.961 [17.673;25.125]
TLCobb	7.200 [3.700;12.800]
baseline_compression	
• No	187 (74.502%)
• Yes	64 (25.498%)
Progressive compression fracture	
• No progression	232 (92.430%)
• Progressive or new-onset compression fracture	19 (7.570%)
Densitometric Results	
aBMD LS (g/cm^2^)	1.019 [0.909;1.145]
aBMD FN (g/cm^2^)	0.800 [0.723;0.879]
aBMD TH (g/cm^2^)	0.604 [0.538;0.692]
T-score LS	−1.140 [−2.002;-0.032]
T-score FN	−1.200 [−1.800;-0.500]
T-score TH	−0.800 [−1.600;0.000]
TBS	1.307 [1.245;1.366]
vBMD from QCT (mg/cm^3^)	81.700 [60.300;103.200]
Diagnosis of LS	
Normal	118 (47.012%)
Osteopenia	106 (42.231%)
Osteoporosis	27 (10.757%)
Diagnosis of FN	
Normal	111 (44.223%)
Osteopenia	114 (45.418%)
Osteoporosis	26 (10.359%)
Diagnosis of TH	
Normal	144 (57.371%)
Osteopenia	86 (34.263%)
Osteoporosis	21 (8.367%)
Status of TBS	
Normal	81 (32.271%)
Partially degraded	127 (50.598%)
Degraded	43 (17.131%)
FRAX	
FRAX major	5.500 [4.250;7.800]
FRAX hip	1.300 [0.500;2.500]
TBS adjusted FRAX major	6.000 [4.450;8.450]
TBS adjusted FRAX hip	1.200 [0.500;2.700]
Diagnosis of QCT	
Normal	36 (14.343%)
Osteopenia	94 (37.450%)
Osteoporosis	121 (48.207%)

BMI: Body mass index, aBMD: Areal bone mineral density, LS: Lumbar spine, FN: Femoral neck, TH: Total hip, TBS: Trabecular bone score, vBMD: Volumetric bone mineral density, QCT: Quantitative computed tomography, FRAX: Fracture Risk Assessment Tool, TLCobb: Cobb angle of the thoracolumbar spine. Variables are expressed as the median (interquartile range).

### Densitometric findings in relation to the presence of compression fracture

No significant differences in mean age or BMI were observed between participants with and without baseline compression fractures. The median TBS at baseline was significantly higher in participants without vertebral fractures than in those with vertebral fractures (1.327 [IQR: 1.268–1.374] vs. 1.252 [IQR: 1.176–1.313], respectively; *p* < 0.001). Participants were diagnosed with degraded microarchitecture based on TBS as follows: participants without baseline compression fracture (n = 19, 10.160%) and participants with baseline compression fractures (n = 24, 37.5%), respectively. All FRAX values were significantly higher in the group with baseline compression fractures (Table 2). A significantly higher proportion of participants with baseline compression fractures showed normal LS, FN, and TH status on DXA compared to normal TBS and QCT (Fig 1).

**Table 2:** Difference in clinical results according to the baseline compression fracture.

	No baseline compression fracture	Baseline compression fracture	p – value
	(N = 187)	(N = 64)	
Height	158.000 [154.000;161.000]	154.450 [150.000;159.000]	<0.001
Weight	59.000 [52.500;64.000]	55.000 [50.000;60.500]	0.003
BMI	24.034 [21.939;25.559]	23.276 [21.083;25.204]	0.197
TLCobb	6.700 [3.350;10.400]	12.200 [5.700;22.850]	<0.001
Progressive compression fracture			0.011
• No progression	178 (95.187%)	54 (84.375%)	
• Progressive or new-onset compression fracture	9 (4.813%)	10 (15.625%)	
Densitometric Results			
aBMD LS (g/cm^2^)	1.036 [0.944;1.157]	0.940 [0.858;1.094]	<0.001
aBMD FN (g/cm^2^)	0.820 [0.790; 0.935]	0.742 [0.708;0.875]	<0.001
aBMD TH (g/cm^2^)	0.620 [0.564;0.712]	0.558 [0.492;0.653]	<0.001
TBS	1.327 [1.268;1.374]	1.252 [1.176;1.313]	<0.001
vBMD from QCT (mg/cm^3^)	83.700 [68.475;105.050]	60.650 [42.600;101.000]	0.002
Diagnosis of LS			0.002
Normal	96 (51.337%)	22 (34.375%)	
Osteopenia	78 (41.711%)	28 (43.750%)	
Osteoporosis	13 (6.952%)	14 (21.875%)	
Diagnosis of FN			<0.001
Normal	95 (50.802%)	16 (25.000%)	
Osteopenia	83 (44.385%)	31 (48.438%)	
Osteoporosis	9 (4.813%)	17 (26.562%)	
Diagnosis of TH			<0.001
Normal	120 (64.171%)	24 (37.500%)	
Osteopenia	60 (32.086%)	26 (40.625%)	
Osteoporosis	7 (3.743%)	14 (21.875%)	
Status of TBS			<0.001
Normal	71 (37.968%)	10 (15.625%)	
Partially deteriorated	97 (51.872%)	30 (46.875%)	
Deteriorated	19 (10.160%)	24 (37.500%)	
Diagnosis of QCT			0.004
Normal	25 (13.369%)	11 (17.188%)	
Osteopenia	81 (43.316%)	13 (20.313%)	
Osteoporosis	81 (43.316%)	40 (62.500%)	
FRAX			
FRAX major	5.000 [4.000;6.800]	7.700 [5.400;11.000]	<0.001
FRAX hip	0.900 [0.400;1.950]	2.450 [1.050;4.700]	<0.001
TBS adjusted FRAX major	5.500 [4.150;7.400]	8.500 [6.000;13.000]	<0.001
TBS adjusted FRAX hip	0.900 [0.400;2.000]	2.800 [1.250;5.500]	<0.001

BMI: Body mass index, aBMD: Areal bone mineral density, LS: Lumbar spine, FN: Femoral neck, TH: Total hip, TBS: Trabecular bone score, vBMD: Volumetric bone mineral density, QCT: Quantitative computed tomography, FRAX: Fracture Risk Assessment Tool, TLCobb: Cobb angle of the thoracolumbar spine.

Variables are expressed as the median (interquartile range).

**Fig 1:**
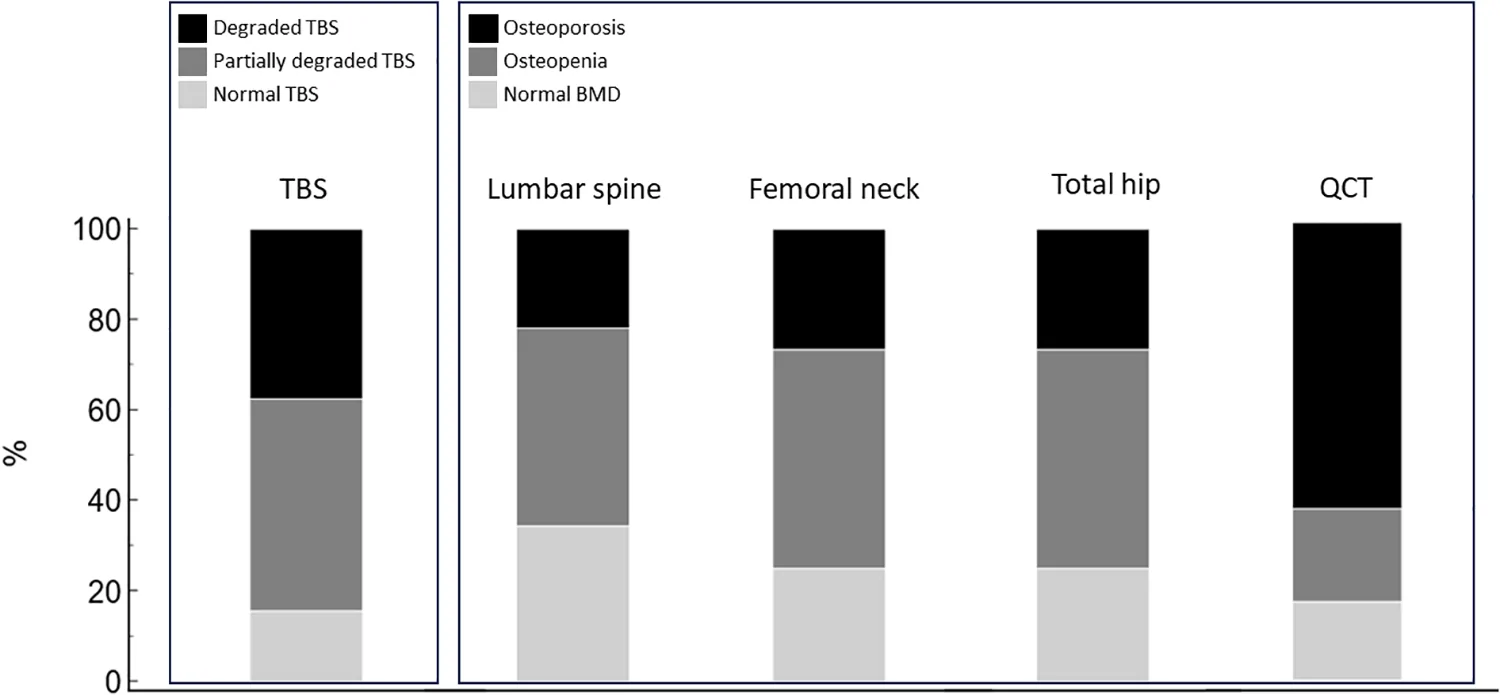
Distribution of bone status according to TBS, DXA, and QCT classifications. The stacked bar graphs show the distribution of participants according to trabecular bone score (TBS), areal bone mineral density (BMD) at the lumbar spine, femoral neck, and total hip, and volumetric BMD assessed by quantitative computed tomography (QCT). TBS was classified as normal, partially degraded, or degraded, while BMD-based assessments were categorized as normal BMD, osteopenia, or osteoporosis. Among participants with baseline compression fractures, a significantly greater proportion were classified as having normal bone status by DXA at the lumbar spine and femur, whereas TBS and QCT more frequently indicated deteriorated bone status.

### Correlations between TLCobb angle, BMD-related values, and FRAX

The TLCobb angle was significantly correlated with TBS-adjusted FRAX. Significant correlations were observed between aBMD, TBS, and TLCobb values at the LS. The TLCobb angle was not significantly associated with femoral BMD parameters, whereas it showed a significant negative correlation with TBS. Using multiple regression tests to determine the FRAX values at the hip, TBS values were significantly correlated with all FRAX values, with and without adjustment for TBS (Fig 2).

**Fig 2:**
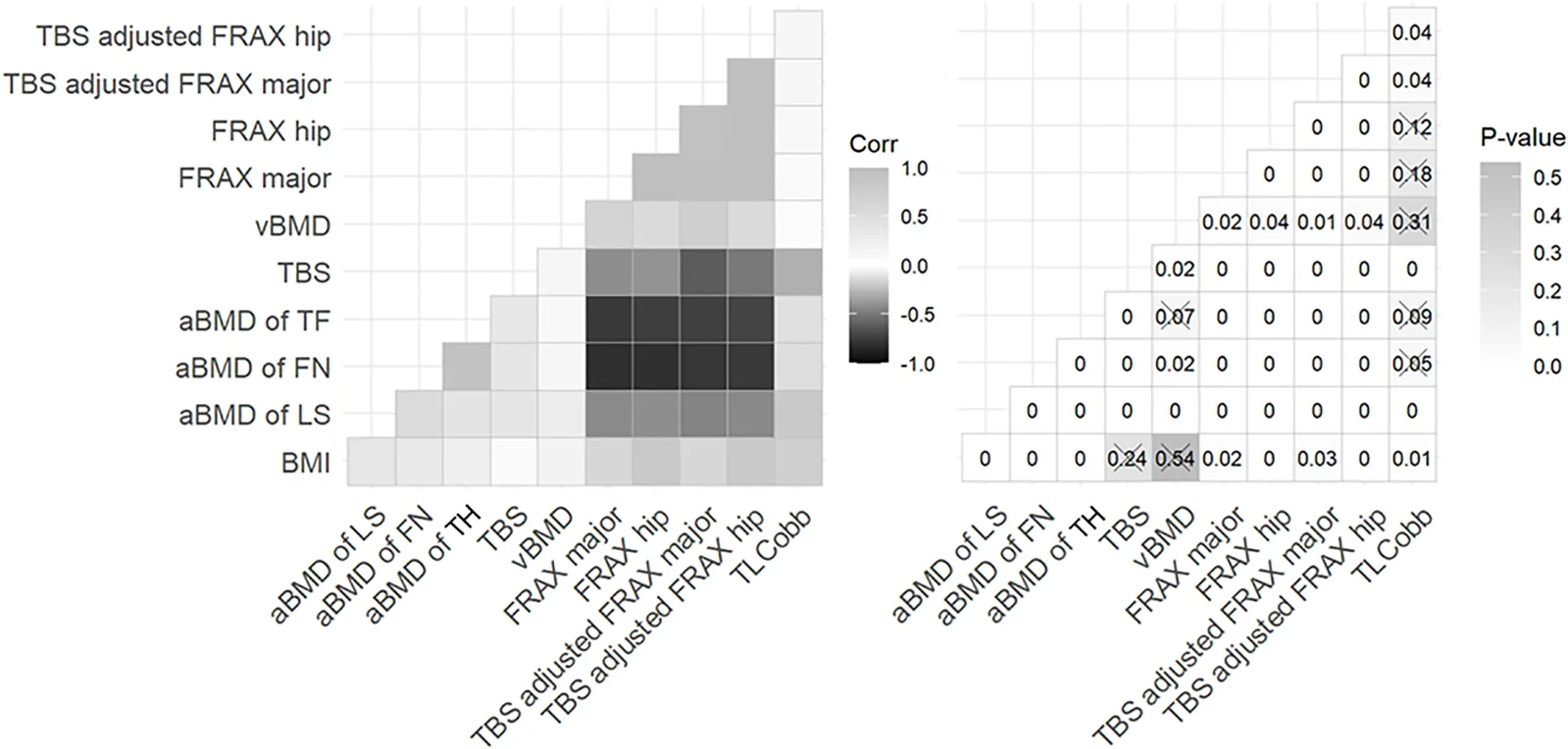
Correlations among TLK, bone density parameters, TBS, and FRAX estimates. Correlation matrix showing the relationships between thoracolumbar Cobb angle (TLCobb) and areal bone mineral density (aBMD) at the lumbar spine (LS), femoral neck (FN), and total hip (TH), volumetric bone mineral density (vBMD), trabecular bone score (TBS), FRAX estimates for major osteoporotic fracture and hip fracture, and TBS-adjusted FRAX estimates. TLCobb was significantly associated with TBS-adjusted FRAX estimates and TBS, but not with femoral BMD parameters, whereas TBS was significantly correlated with all FRAX estimates, both before and after TBS adjustment. (A) Correlation coefficients are displayed using a graded scale, with darker shading indicating stronger correlations. (B) Corresponding P values for each pairwise correlation are shown.

Notably, the TLCobb angle was not significantly associated with femoral BMD parameters, whereas it showed a significant correlation with TBS and FRAX-related risk estimates, indicating that increased TLK is linked to impaired bone quality and higher fracture risk despite relatively preserved or even increased LS BMD.

### Predictive factors including densitometric markers of fragility fracture-free survival

Nineteen patients experienced progressive or new-onset vertebral compression fractures during a median follow-up of 20 months (range, 12–32 months). The threshold for the TLCobb angle was set at 9.4° using ROC analysis (area under the curve, 0.702, *p* = 0.001). Univariate analyses revealed that baseline compression fractures; diagnosis of osteoporosis based on T-scores or vBMD measurements at the LS, FN, and TH; and degraded microarchitecture based on TBS and TLCobb angle values above 9.4° were significant predictors of fracture-free survival after initial densitometric assessment (Fig 3). The final multivariate Cox regression analysis showed that baseline compression fractures (hazard ratio [HR]: 3.763, *p* = 0.006) and TLCobb angle values > 9.4° (HR: 4.889, *p* = 0.003) were independent predictive factors for future progressive or new-onset fragility fractures (Table 3).

**Table 3:** Cox proportional hazards model for the fracture.

Covariate	Univariate analysis		Multivariate analysis	
	HR (95% CI)	*P*-value	HR (95% CI)	*P*-value
Baseline compression fracture	4.558 (1.844;11.265)	0.001	3.763 (1.478;9.603)	0.006
T-score of LS ≤ −2.5	3.009 (1.454;6.227)	0.003		
T-score of FN ≤ −2.5	3.657 (1.921;6.960)	0.001		
T-score of TH ≤ −2.5	2.601 (1.338;5.080)	0.003		
vBMD of LS ≤ 80 mg/cm^3^	1.936 (1.091;4.118)	0.006		
TBS ≤ 1.200	3.650 (1.369;6.128)	0.004		
TLCobb > 9.4°	5.35 (2.003;14.305)	0.002	4.889 (1.727;13.841)	0.003

LS: Lumbar spine, FN: Femoral neck, TH: Total hip, vBMD: Volumetric bone mineral density, TBS: Trabecular bone score, TLCobb: Cobb angle of the thoracolumbar spine

**Fig 3:**
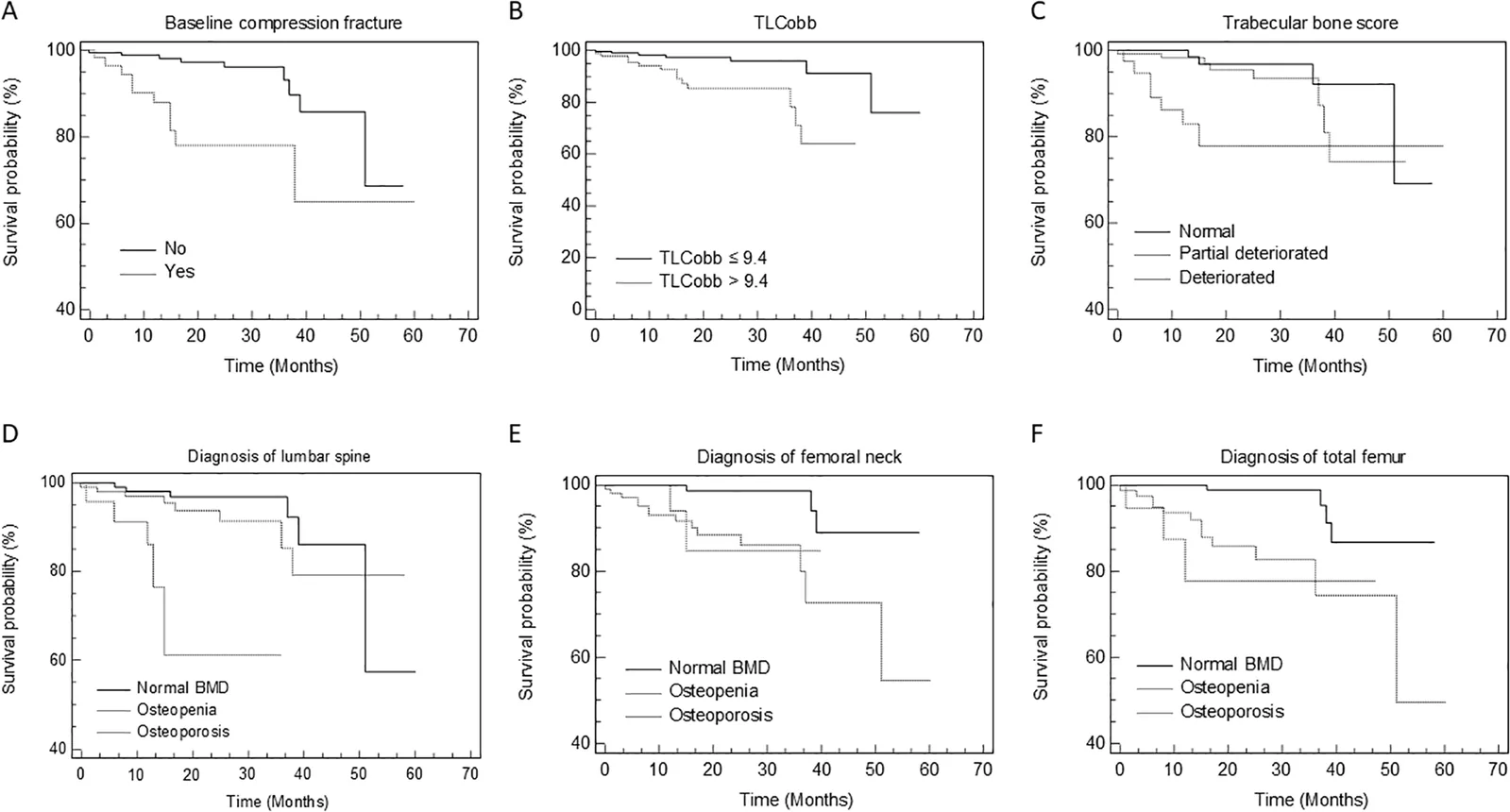
Kaplan–Meier curves for incident fragility fracture according to baseline skeletal and radiographic characteristics. Kaplan–Meier curves showing fracture-free survival according to (A) the presence or absence of baseline vertebral compression fracture, (B) thoracolumbar Cobb angle (TLCobb ≤ 9.4° vs. > 9.4°), (C) trabecular bone score (TBS) category, and bone mineral density (BMD) categories at the (D) lumbar spine, (E) femoral neck, and (F) total hip. TBS was classified as normal, partially degraded, or degraded, whereas BMD was categorized as normal, osteopenic, or osteoporotic. Survival probability represents the probability of remaining free of progressive or new-onset fragility fractures during follow-up.

## Discussion

In this retrospective study, participants with baseline compression fractures exhibited reduced bone mass and quality, with an increased TLCobb angle range. Additionally, they showed a higher proportion of deteriorated bone states based on the TBS or osteoporosis assessed by vBMD. In terms of progressive or new-onset fragility compression fractures, baseline compression fractures and increased TLCobb angles were associated with a statistically significant increase in the HR for future fragility fractures.

Vertebral fractures in patients with osteoporosis are associated with increased mortality, reduced quality of life, and several comorbidities; therefore, primary and secondary prevention of fragility fractures is crucial [17,18]. Although aBMD measured using DXA is used to diagnose osteoporosis and predict fracture risk, many fragility fractures occur in individuals with T-scores derived from aBMD values above the osteoporotic range [19]. Consistent with the results of previous studies, approximately half of the participants with baseline compression fractures in the present study had normal or osteopenic LS BMD T-scores. Overestimation of aBMD at the LS is primarily due to degenerative changes and abdominal aortic calcification. Based on these findings, alternative methods such as TBS and vBMD have been proposed to assess bone quality and microarchitecture. Participants with compression fractures exhibited poorer bone quality, as assessed by TBS and vBMD, compared to aBMD values. These findings are consistent with those of previous studies comparing QCT and DXA [14].

TLK is typically caused by previous vertebral fractures and osteoporosis, which tend to worsen with age [20]. Osteoporotic changes can reduce the biomechanical strength of vertebral bodies, particularly the anterior column [21]. Compressive overload is transmitted to the vertebral bodies in the upright position, which can damage the endplates. Repetitive cyclic loading results in progressive collapse of the unsupported vertebral cortex, contributing to TLK [22]. In addition, decreased lumbodorsal muscle quantity and strength can lead to TLK in older adults with osteoporosis. The clinical significance of TLK lies in its association with osteoporosis and osteoporotic fractures, as well as a notable decline in pulmonary function, which reduces both survival rate and quality of life [23].

Although TLK is a recognized structural consequence of previous fractures, multivariate analysis demonstrates that it also serves as a significant independent predictor of future fractures. This suggests a biomechanical vicious cycle in which initial fractures lead to a kyphotic deformity, representing an increased TLCobb angle, which shifts the body’s center of gravity forward. This shift increases the anterior wedge-loading forces on the adjacent vertebrae, thereby predisposing them to subsequent progressive or new-onset fractures, independent of the densitometric variables included in the model. Thus, an increased TLCobb angle is not merely a manifestation of past skeletal damage but a critical factor in the progression of spinal instability.

FRAX does not provide an absolute measure of fracture risk because it is a tool for assessing risk and cannot be considered fully accurate. Additionally, FRAX values may vary across countries, particularly among older women [24]. All FRAX values exhibited a strong correlation with TBS and BMD but not with TLK. We assumed that this was because FN BMD had a definite effect on FRAX values. However, this effect diminished when FRAX values were adjusted for TBS, resulting in a weaker but significant correlation between TLCobb angle and FRAX. These findings further support the potential role of TLK as a complementary parameter that may improve fracture risk stratification beyond conventional tools such as BMD and FRAX.

In the current study, in postmenopausal women, the future prevalence of progressive or new-onset fractures was found to be strongly associated with the presence of a predisposing compression fracture and the extent of TLK, depicted as a TLCobb angle > 9.4°. Our results help explain the increased risk of secondary vertebral compression fractures in patients with a history of such fractures [25,26]. These findings indicate that women with osteoporosis, TLK, and increased sagittal TLCobb angle should be closely monitored and managed. Our study suggested a TLCobb threshold > 9.4°; however, Wei et al. suggested that cases with a sagittal TLCobb angle > 7.5° should be managed accordingly, and that a sagittal TLCobb angle > 15° should be considered a red flag [27]. The difference in the threshold value is assumed to be due to the fact that the patients included in our study were relatively healthy individuals with no significant medical issues.

Several previous cross-sectional and prospective studies have reported an association between the TBS and vBMD for predicting osteoporotic fractures [28–31]. Furthermore, previous reports have demonstrated that QCT predicts vertebral fractures more effectively than aBMD-defined DXA [32]. In the current study, the diagnostic results from TBS and cQCT could also have improved fracture risk prediction in the univariate analysis; however, degraded microarchitecture, as indicated by TBS and osteoporosis detected on cQCT, was not associated with an increased risk of future fragility vertebral fractures in multivariate analysis. It is assumed that this is due to the high sensitivity of the diagnostic criteria for bone deterioration or osteoporotic changes based on TBS and vBMD, as well as the fact that the included patients were healthy rather than in a pathological state. More reliable results are expected with a combination of aBMD, TBS, and cQCT results, as previous studies have reported a more accurate prediction of fracture risk, regardless of ethnicity [28,30].

This study has several limitations. First, its retrospective, single-center design and the selective use of cQCT based on patient preference or clinical indication may have introduced selection bias and limited the generalizability of the findings. Second, fragility fractures were primarily identified using thoracolumbar spine radiographs; therefore, fractures at other sites, particularly the femoral neck, may have been missed.

Despite these limitations, TLK may provide complementary information beyond conventional fracture risk assessments. DXA, QCT, and FRAX primarily reflect bone mass and established clinical risk factors but do not fully capture vertebral deformity, sagittal alignment, or altered spinal loading. Because fragility fractures may occur even in individuals without osteoporotic BMD, TLK may serve as an additional radiographic marker to refine fracture risk stratification in postmenopausal women.

In conclusion, increased TLK, as measured using the thoracolumbar Cobb angle, is associated with future vertebral compression fracture risk in postmenopausal Korean women. Baseline compression fractures and a TLCobb angle > 9.4° were independent predictors of progressive or new-onset fragility compression fractures. These findings suggest that TLK may serve as a simple adjunctive radiographic marker for refining fracture risk stratification, complementary to conventional BMD and FRAX assessment.
